# Comparison of Short- vs Long-axis Technique for Ultrasound-guided Peripheral Line Placement: A Systematic Review and Meta-analysis

**DOI:** 10.7759/cureus.2718

**Published:** 2018-05-31

**Authors:** Michael Gottlieb, Dallas Holladay, Gary D Peksa

**Affiliations:** 1 Department of Emergency Medicine, Rush University Medical Center, Chicago, USA; 2 Pharmacy and Emergency Medicine, Rush University Medical Center, Chicago, USA

**Keywords:** ultrasound, peripheral intravenous line, peripheral iv, short-axis, long-axis, emergency medicine, systematic review

## Abstract

Ultrasound-guided peripheral intravenous line (PIV) placement is associated with increased success rates, decreased time to cannulation, and fewer skin punctures than traditional, landmark-based techniques. However, it is unclear which technique is best. This review compares the short-axis (SA) and long-axis (LA) techniques for PIV placement.

PubMed, Embase, Scopus, the Cochrane Database of Systematic Reviews, the Cochrane Central Register of Controlled Trials, and bibliographies of selected articles were assessed for prospective trials evaluating the first pass success rate of SA vs LA ultrasound-guided PIV placement. Secondary outcomes included time to placement, number of needle passes, and incidence of posterior wall puncture. Data were double extracted into a predefined worksheet and quality was assessed using the Cochrane Risk of Bias tool.

Three studies (n = 198) were identified. SA was successful in 125/128 placements (97.7%) and LA technique was successful in 114/128 placements (89.1%). There was an odds ratio of 5.35 (95% CI: 1.46-19.58) in favor of the SA technique. There was no difference in the mean number of needle passes. Time to insertion varied between studies.

The existing literature suggests that the SA technique is associated with greater success than the LA technique. Based upon the data, short-axis may be considered as the first approach for ultrasound-guided PIV placement among providers comfortable with both techniques, though further studies are needed.

## Introduction and background

Peripheral intravenous line (PIV) placement is one of the most commonly performed procedures in the Emergency Department (ED) [1]. Traditional, landmark-based PIV placement has a success rate of approximately 91% in the emergency setting [2]. However, certain medical conditions such as diabetes, sickle cell disease, and intravenous drug use are associated with increased difficulty with PIV placement [3]. Failed landmark-based PIV placement has previously required central venous or intraosseous access, which can be associated with an increased risk of complications [4]. Recently, there has been a growing body of literature supporting the use of ultrasound-guided PIV placement among ED nurses, technicians, and physicians [5-8]. Multiple studies have demonstrated that ultrasound-guided PIV access is associated with increased success rates, decreased time to cannulation, and fewer skin punctures [5-7,9]. Although studies have shown increased success with ultrasound-guided PIVs, there is currently no standard method for insertion. Some users favor the longitudinal, in-plane approach whereas others prefer the short axis, out-of-plane technique. The aim of this paper was to perform a systematic review and meta-analysis of existing trials that compare the short- and long-axis techniques for ultrasound-guided PIV placement.

## Review

Methods

This protocol (#CRD42017073249) was registered with and is available for review at the PROSPERO website (https://www.crd.york.ac.uk/PROSPERO/). Our study conforms to the Preferred Reporting Items for Systematic Reviews and Meta-Analyses (PRISMA) guidelines for systematic reviews and was performed in accordance with best practice guidelines [10]. In conjunction with a medical librarian, we conducted a search of PubMed, Embase, Scopus, the Cochrane Database of Systematic Reviews, and the Cochrane Central Register of Controlled Trials to include citations from inception to August 4, 2017. Details of the search strategy are included in the Appendix. We reviewed the bibliographies of identified studies and review articles for potential missed articles. We also consulted with topic experts to help identify any further relevant studies.

Inclusion criteria consisted of all prospective, observational and randomized, controlled trials assessing the first pass success rates of the short- versus long-axis approach for ultrasound-guided PIV placement. Secondary outcomes included time to placement, number of needle passes, and incidence of posterior wall puncture. Exclusion criteria included retrospective studies, case series, and studies published in abstract format only. There were no language exclusions. Two physician-investigators independently assessed studies for eligibility based upon the above criteria. All abstracts meeting initial criteria were reviewed as full manuscripts. Studies determined to meet the eligibility criteria on full text review by both extractors were included in the final data analysis. Any discrepancies were resolved by consensus.

Two physician-investigators independently extracted data from the included studies. The investigators underwent initial training and extracted data into a pre-designed data collection form. The following information was abstracted: last name of the first author, study title, publication year, total study population size, study country, study location, use of a phantom model versus live patient, sonographer experience, ultrasound training protocol, type of PIV catheter used, confirmation method, first pass success rate, number of needle passes, rates of posterior wall puncture, and time to placement. Studies were independently assessed for quality by two separate physician-investigators utilizing the Cochrane Risk of Bias tool [11]. Any discrepancies were resolved by consensus.

The difference in success rates was measured by an odds ratio (OR) with a 95% confidence interval (CI). The pooled data were analyzed using a random effects model and the Mantel-Haenszel method. The mean number of needle passes were assessed for mean difference with a 95% CI. The pooled data were analyzed using a random effects model and the inverse-variance method. For all pooled analyses, a two-sided p-value < 0.05 was considered statistically significant. Each study used a different reporting strategy for insertion time, limiting the ability to perform a meta-analysis on this data set. The authors of the studies were contacted for access to the original data, but it was no longer available. Therefore, insertion time is reported only in a qualitative manner. Chi-square and I^2^ statistics were utilized to assess heterogeneity of included studies, with a p-value < 0.1 or I^2^ > 50% considered significant for heterogeneity. We utilized a funnel plot and Egger’s test to assess for publication bias. All analyses were performed using RevMan (The Nordic Cochrane Centre, Copenhagen, Denmark), version 5.3.

Results

**Figure 1:**
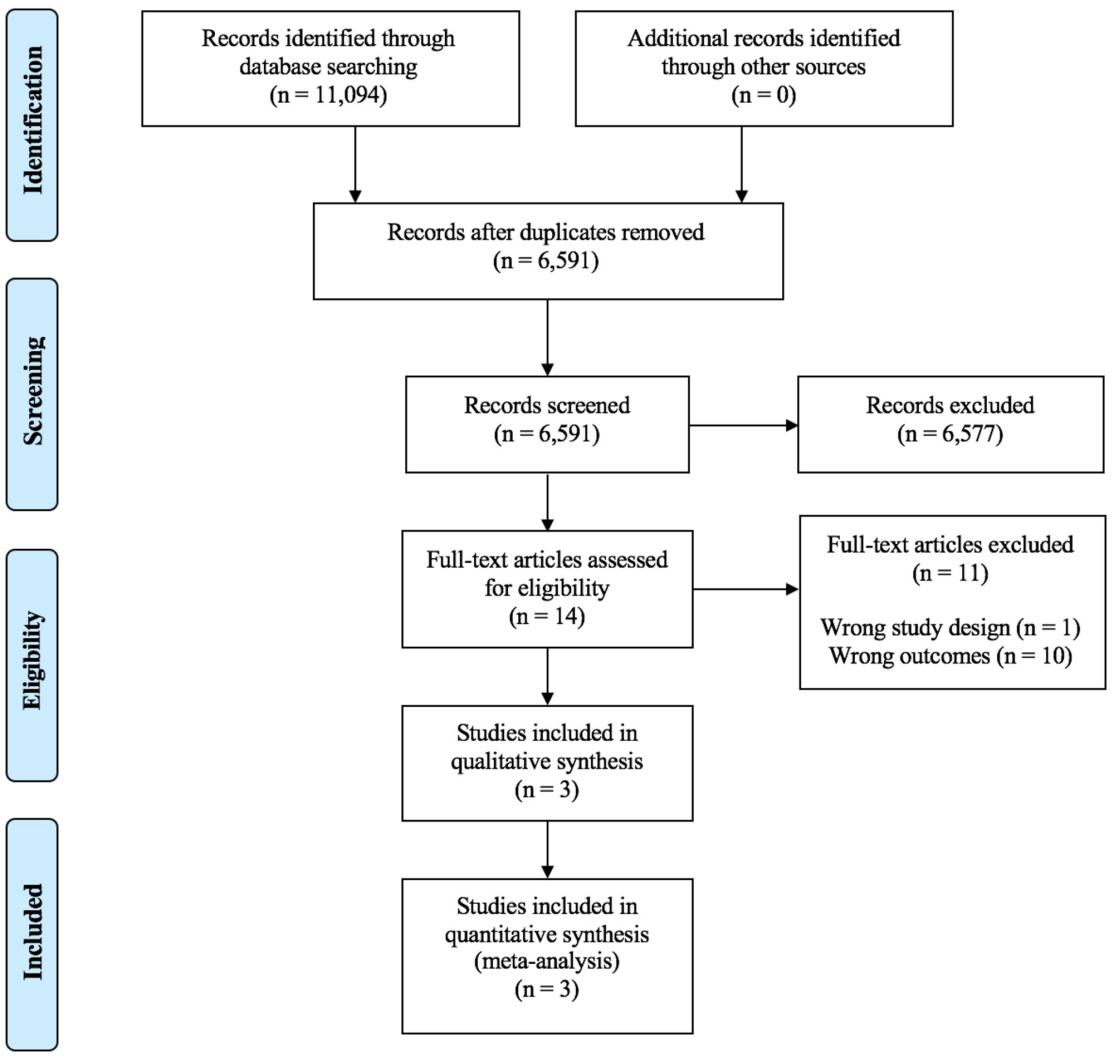
PRISMA diagram. PRISMA: Preferred reporting items for systematic reviews and meta-analyses

A total of 11,094 studies were identified. PubMed yielded 7,858, Scopus identified 3,132, the Cochrane Database of Systematic Reviews found one article, and the Cochrane Central Register of Controlled Trials yielded 103 studies. After removal of duplicated, 6,591 abstracts were screened with 14 selected for full text review (Figure 1). No additional papers were identified through bibliographic review.

Three studies, comprising 198 total assessments, were selected for the final analysis (Table 1). There was one prospective, observational study [12] and two randomized, controlled trials [13,14]. Two studies were performed on a phantom model [12,14] and one was performed in live patients [13].

**Table 1:** Summary of existing studies comparing short- versus long-axis technique for peripheral line placement. RCT: Randomized controlled trial; US: Ultrasound; PIV: Peripheral intravenous line.

Study	Phantom or Live Patient	Study Design	Sonographer Experience	Ultrasound Training Protocol	Study Population Size	Peripheral Catheter Length and Gauge	Catheter Confirmation Technique
Mahler 2011 [13]	Live Patient	RCT	Emergency physicians and nurses with prior US-guided PIV experience	30 minutes of lecture and one hour of hands-on time with vascular access phantom	40	4.45-cm; 18- or 20-gauge	Blood return through PIV and saline flush test using power Doppler
Clemmesen 2012 [14]	Phantom	RCT	Novice sonographers	One hour of lecture and one hour of hands-on time with vascular access phantom	58	18-gauge (length not specified)	Expert visualization of needle within the vessel on ultrasound
Erickson 2014 [12]	Phantom	Prospective, observational	Novice sonographers (nurses with no prior US experience)	One hour of lecture followed by hands-on scanning time and 2 practice attempts with the vascular access phantom	100	Not described	Blood return through PIV and needle visualized on ultrasound

**Figure 2:**
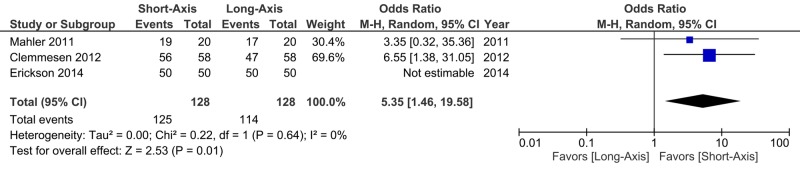
Difference in success rates between the short-axis and long-axis techniques.

**Figure 3:**
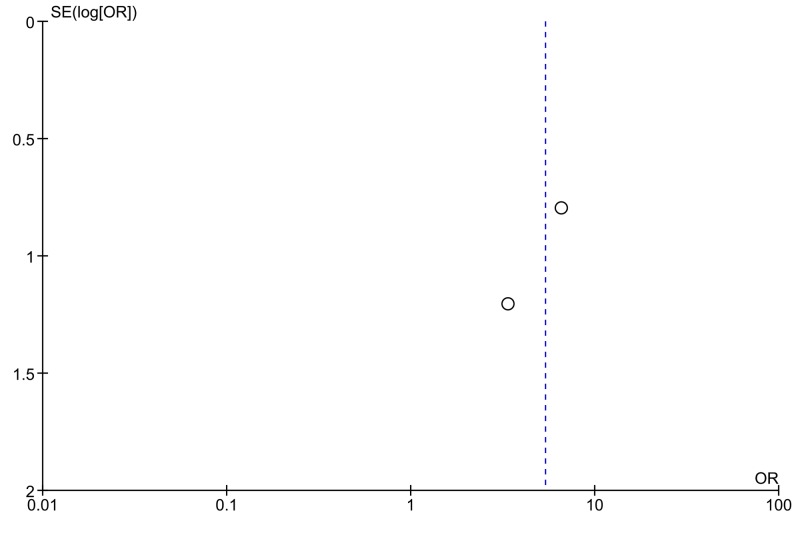
Funnel plot.

Overall, the short-axis technique was successful in 125 of 128 total placements (97.7%). The long-axis technique was successful in 114 of 128 total placements (89.1%). There was an OR of 5.35 (95% CI: 1.46 to 19.58) in favor of the short-axis technique (Figure 2). There was no evidence of statistical heterogeneity with an I^2^ of 0%. Funnel plot analysis demonstrated no evidence of publication bias, though assessment was limited by the number of studies (Figure 3).

The mean number of needle passes was not significantly different between the short- and long-axis techniques with a mean difference of 0.10 needle passes (95% CI: -0.33 to 0.53). The insertion time could not be quantitatively combined due to differences in reporting strategy. Mahler et al. identified a statistically significant reduction in time for the short-axis technique [13], while Clemmesen et al. identified a statistically significant reduction in time for the long-axis technique (Table 2) [14]. Erickson et al. did not identify a significant difference between either technique [12]. No studies assessed rates of posterior wall puncture for PIV placement.

**Table 2:** Comparison of insertion times between short- versus long-axis technique for peripheral line placement. ^A ^Median (Interquartile Range); ^B^ Mean (Standard Deviation); ^C^ Median (95% Confidence Interval)

Study	Insertion Time for Short-Axis (seconds)	Insertion Time for Long-Axis (seconds)
Mahler 2011 [13]	34 (35)^A^	96 (59)^A^
Clemmesen 2012 [14]	36 (31)^B^	22 (17)^B^
Erickson 2014 [12]	10 (6-13)^C^	11 (7-12)^C^

Studies were at overall low risk of bias (Tables 3, 4). The prospective, observational study by Erickson et al. was at low risk of bias for all criteria [12]. The randomized controlled trials were at low risk of bias for all criteria except for unclear blinding of participants and personnel in one study [13] and unclear blinding of outcome assessment in both studies [13,14].

**Table 3:** Assessment of study quality for prospective studies. L: Low risk of bias

Study	Confounding	Selection of Participants	Measurement of Interventions	Departures from Intended Interventions	Missing Data	Measurement of Outcomes	Selection of Reported Results
Erickson 2014 [12]	L	L	L	L	L	L	L

**Table 4:** Assessment of study quality for the randomized, controlled trial. L: Low risk of bias; U: Unclear risk of bias.

Study	Random Sequence Generation	Allocation Concealment	Selective Reporting	Other Bias	Blinding of Participants and Personnel	Blinding of Outcome Assessment	Incomplete Outcome Data
Mahler 2011 [13]	L	L	L	L	U	U	L
Clemmesen 2012 [14]	L	L	L	L	L	U	L

Discussion

To the best of our knowledge, this is the first systematic review and meta-analysis comparing the short-axis versus the long-axis technique specifically for peripheral intravenous line placement. Overall, this study demonstrated that the short-axis approach was associated with a greater likelihood of successful placements when compared with the long-axis.

Gao et al. performed a systematic review and meta-analysis comparing the short-axis with the long-axis technique for all vascular access in 2016 and was unable to identify a significant difference between the techniques for first pass success, mean placement time, or number of attempts [15]. However, that review was performed two years prior to the current study with limited search criteria and, consequently, only identified one trial comparing these techniques for peripheral line placement. Our current review was able to identify two additional studies, which allowed for greater power to detect a difference in success rates.

We believe that it is important to separate out PIV placement from other forms of vascular access (e.g., central venous access, arterial access), as the skills involved in peripheral intravenous access differ significantly from other techniques. While central venous and arterial access are performed using a Seldinger technique, peripheral line placement requires advancement of the catheter without the assistance of a guidewire, which may be more challenging for many providers [16]. As such, we sought to intentionally determine the effectiveness, number of needle passes, and time to placement among this specific group.

When performed for peripheral venous access, the long-axis technique has been suggested to be superior due to the ability to completely visualize the entire length of the catheter to avoid accidental posterior wall punctures [17,18]. However, it may be challenging to maintain both the vessel and catheter in the same plane, leading many providers to favor the short-axis technique [16]. Based upon the current data, the short-axis technique was associated with greater success rates than the long-axis technique.

Interestingly, there was no significant difference in the number of needle passes between groups. Because there was a low number of needle passes with each technique, it is possible that the studies were underpowered to detect a significant difference. However, given the low overall number, any statistically significant difference between studies would be unlikely to be clinically significant.

We were unable to perform a meta-analysis of the difference in insertion times due to variations in reporting and lack of access to the original data. One study demonstrated an almost three-fold increase in insertion times with the long-axis technique [13], while another demonstrated a 60% increase in insertion times with the short-axis technique [14].

It is important to consider several limitations with respect to this review. Overall, there were a limited number of studies, which comprised only 198 total assessments. Additionally, two of the studies were performed in a vascular access phantom, which may not completely replicate live tissue. However, the success rates were not significantly different between the live patients and vascular access phantoms, with both favoring the short-axis technique. Furthermore, this study only assessed peripheral vascular access and intentionally excluded central venous and arterial access. However, the latter two techniques utilize a Seldinger approach, which may not require as precise identification of the needle tip within the vessel. As the intention of this study was to specifically assess PIV placement, studies using large bore needles for aspiration were excluded. Finally, we were unable to combine the data for insertion times due to differences in reporting methods and lack of access to the original data. Therefore, it is not possible to reliably determine which technique was more rapid to perform.

Further studies are recommended to assess differences in insertion times between techniques to ascertain which is more efficient. Future studies should also compare the short-axis versus the long-axis technique in a larger sample of live patients, as well as among providers of varying levels of training.

## Conclusions

The existing literature suggests that the short-axis technique is associated with a greater success rate than the long-axis technique. Based upon the data, short-axis may be considered as the first approach for ultrasound-guided PIV placement among providers comfortable with both techniques, though further studies on live patients are needed.

## Appendices

**Table 5:** Search strategy.

Cochrane Central Register of Controlled Trials	Number of Search Results	Search Strategies	Number of Search Results	
(ZE "ultrasonography")	111 results	(ZE "prospective studies") OR (ZE "randomized controlled trial") OR (ZE "controlled clinical trial") AND (ZE "ultrasonography") OR "ultrasound-guided peripheral intravenous access"	0 results	
(ZE "emergency medicine")	14 results	(ZE "prospective studies") OR (ZE "randomized controlled trial") OR (ZE "controlled clinical trial") AND (ZE "ultrasonography") OR "ultrasound-guided peripheral intravenous access" AND (ZE "emergency medicine"))	0 results	
(ZE "prospective studies")	9 results			
(ZE "randomized controlled trial")	13 results			
(ZE "controlled clinical trial")	1 results			
ultrasound-guided peripheral intravenous access	13 results			
ultrasound-guided peripheral intravenous access, Limiter-Randomized Controlled Trials	7 results			
(ZE "prospective studies") OR (ZE "randomized controlled trial") OR (ZE "controlled clinical trial")	22 results			
PubMed				
Keywords	MeSH	Search Strategies	Final Search Strategy	Number of Search Results
"in-plane"	N/A	(("long axis"[text word] OR "in-plane"[text word]) OR ("short axis"[text word] OR "out-of-plane"[text word]))	((((("long axis"[text word] OR "in-plane"[text word]) OR ("short axis"[text word] OR "out-of-plane"[text word]))))) AND (((ultrasound*[text word] OR ultrasonography[text word] OR "ultrasound imaging"[text word] OR "Imaging, Ultrasound"[text word] OR "Imagings, Ultrasound"[text word] OR "Ultrasound Imagings"[text word] OR "Sonography, Medical"[text word] OR "Medical Sonography"[text word] OR "Ultrasonography"[Mesh] OR "ultrasound-guided peripheral"[text word] OR "ultrasound-guided peripheral intravenous access"[text word] OR "ultrasound-guided vascular access"[text word] OR "ultrasound-guided venous access"[text word] OR USGVA OR "ultrasound-guided small vessel penetration"[text word] OR "ultrasound-guided small vessel penetration"[text word] OR "ultrasound guidance"[text word])))	4040
"out-of-plane"	N/A			
"long axis"	N/A			
"short axis"	N/A			
ultrasound	N/A			
ultrasonography ultrasound imaging Imaging, Ultrasound Imagings, Ultrasound Ultrasound Imagings Sonography, Medical Medical Sonography	"Ultrasonography"[Mesh]	(ultrasound*[text word] OR ultrasonography[text word] OR "ultrasound imaging"[text word] OR "Imaging, Ultrasound"[text word] OR "Imagings, Ultrasound"[text word] OR "Ultrasound Imagings"[text word] OR "Sonography, Medical"[text word] OR "Medical Sonography"[text word] OR "Ultrasonography"[Mesh] OR "ultrasound-guided peripheral"[text word] OR "ultrasound-guided peripheral intravenous access"[text word] OR "ultrasound-guided vascular access"[text word] OR "ultrasound-guided venous access"[text word] OR USGVA OR "ultrasound-guided small vessel penetration"[text word] OR "ultrasound-guided small vessel penetration"[text word] OR "ultrasound guidance"[text word])		
ultrasound-guided peripheral	N/A		(((((((("long axis"[text word] OR "in-plane"[text word]) OR ("short axis"[text word] OR "out-of-plane"[text word]))))) AND (((ultrasound*[text word] OR ultrasonography[text word] OR "ultrasound imaging"[text word] OR "Imaging, Ultrasound"[text word] OR "Imagings, Ultrasound"[text word] OR "Ultrasound Imagings"[text word] OR "Sonography, Medical"[text word] OR "Medical Sonography"[text word] OR "Ultrasonography"[Mesh] OR "ultrasound-guided peripheral"[text word] OR "ultrasound-guided peripheral intravenous access"[text word] OR "ultrasound-guided vascular access"[text word] OR "ultrasound-guided venous access"[text word] OR USGVA OR "ultrasound-guided small vessel penetration"[text word] OR "ultrasound-guided small vessel penetration"[text word] OR "ultrasound guidance"[text word])))))) AND (("peripheral IV"[text word] OR "peripheral intravenous cannulation"[text word] OR "catheterization of vein and artery"[text word] OR "vascular cannulation"[text word] OR "cannulated vessels"[text word] OR "landmark techniques"[text word] OR cannula[text word] OR cannulae[text word] OR "nasal cannula"[text word] OR "Cannula"[Mesh] OR "Catheters"[Mesh] OR vein[text word] OR "Veins"[Mesh] OR insertion[text word] OR "vessel penetration"[text word] OR "skin penetration"[text word] OR "catheter redirection"[text word] OR "Vascular Access Devices"[Mesh] OR "Catheters"[Mesh] OR "Catheterization, Peripheral"[Mesh]))	436
ultrasound-guided peripheral intravenous access	N/A			
ultrasound-guided vascular access	N/A			
ultrasound-guided venous access	N/A			
USGVA	N/A			
ultrasound-guided small vessel penetration	N/A			
ultrasound guidance	N/A			
peripheral IV	N/A	"peripheral IV"[text word] OR "peripheral intravenous cannulation"[text word] OR "catheterization of vein and artery"[text word] OR "vascular cannulation"[text word] OR "cannulated vessels"[text word] OR "landmark techniques"[text word] OR cannula[text word] OR cannulae[text word] OR "nasal cannula"[text word] OR "Cannula"[Mesh] OR "Catheters"[Mesh] OR vein[text word] OR "Veins"[Mesh] OR insertion[text word] OR "vessel penetration"[text word] OR "skin penetration"[text word] OR "catheter redirection"[text word] OR "Vascular Access Devices"[Mesh] OR "Catheters"[Mesh] OR "Catheterization, Peripheral"[Mesh]		
peripheral intravenous cannulation	N/A			
"out-of-plane"	N/A			
catheterization of vein and artery	N/A			
vascular cannulation	N/A			
cannulated vessels	N/A			
landmark techniques	N/A			
cannula cannulae nasal cannula	"Cannula"[Mesh] "Catheters"[Mesh]			
vein	"Veins"[Mesh]			
insertion	N/A			
vessel penetration	N/A			
skin penetration	N/A			
catheter redirection	"Vascular Access Devices"[Mesh] "Catheters"[Mesh] "Catheterization, Peripheral"[Mesh]			
emergency department emergency services, hospital hospital emergency services services, hospital emergency emergency units	"Emergency Service, Hospital"[Mesh]	("emergency department"[text word] OR "emergency services, hospital"[text word] OR "hospital emergency services"[text word] OR "services, hospital emergency"[text word] OR "Emergency Service, Hospital"[Mesh] OR "emergency units"[text word] OR "emergency medicine"[text word] OR "Medicine, Emergency"[text word] OR "Emergency Medicine"[Mesh])	Search ((((((((((("long axis"[text word] OR "in-plane"[text word]) OR ("short axis"[text word] OR "out-of-plane"[text word]))))) AND (((ultrasound*[text word] OR ultrasonography[text word] OR "ultrasound imaging"[text word] OR "Imaging, Ultrasound"[text word] OR "Imagings, Ultrasound"[text word] OR "Ultrasound Imagings"[text word] OR "Sonography, Medical"[text word] OR "Medical Sonography"[text word] OR "Ultrasonography"[Mesh] OR "ultrasound-guided peripheral"[text word] OR "ultrasound-guided peripheral intravenous access"[text word] OR "ultrasound-guided vascular access"[text word] OR "ultrasound-guided venous access"[text word] OR USGVA OR "ultrasound-guided small vessel penetration"[text word] OR "ultrasound-guided small vessel penetration"[text word] OR "ultrasound guidance"[text word])))))) AND (("peripheral IV"[text word] OR "peripheral intravenous cannulation"[text word] OR "catheterization of vein and artery"[text word] OR "vascular cannulation"[text word] OR "cannulated vessels"[text word] OR "landmark techniques"[text word] OR cannula[text word] OR cannulae[text word] OR "nasal cannula"[text word] OR "Cannula"[Mesh] OR "Catheters"[Mesh] OR vein[text word] OR "Veins"[Mesh] OR insertion[text word] OR "vessel penetration"[text word] OR "skin penetration"[text word] OR "catheter redirection"[text word] OR "Vascular Access Devices"[Mesh] OR "Catheters"[Mesh] OR "Catheterization, Peripheral"[Mesh])))) AND ((("emergency department"[text word] OR "emergency services, hospital"[text word] OR "hospital emergency services"[text word] OR "services, hospital emergency"[text word] OR "Emergency Service, Hospital"[Mesh] OR "emergency units"[text word] OR "emergency medicine"[text word] OR "Medicine, Emergency"[text word] OR "Emergency Medicine"[Mesh])))) OR (((sonographers[text word] OR "emergency department sonographer"[text word] OR "emergency medicine nurse"[text word] OR "emergency department patient"[text word])))	1281
emergency medicine Medicine, Emergency	"Emergency Medicine"[Mesh]			
sonographers	N/A			
emergency department sonographer	N/A			
emergency medicine nurse	N/A			
emergency department patient	N/A			
	N/A	(sonographers[text word] OR "emergency department sonographer"[text word] OR "emergency medicine nurse"[text word] OR "emergency department patient"[text word])	((((((((((((("long axis"[text word] OR "in-plane"[text word]) OR ("short axis"[text word] OR "out-of-plane"[text word]))))) AND (((ultrasound*[text word] OR ultrasonography[text word] OR "ultrasound imaging"[text word] OR "Imaging, Ultrasound"[text word] OR "Imagings, Ultrasound"[text word] OR "Ultrasound Imagings"[text word] OR "Sonography, Medical"[text word] OR "Medical Sonography"[text word] OR "Ultrasonography"[Mesh] OR "ultrasound-guided peripheral"[text word] OR "ultrasound-guided peripheral intravenous access"[text word] OR "ultrasound-guided vascular access"[text word] OR "ultrasound-guided venous access"[text word] OR USGVA OR "ultrasound-guided small vessel penetration"[text word] OR "ultrasound-guided small vessel penetration"[text word] OR "ultrasound guidance"[text word])))))) AND (("peripheral IV"[text word] OR "peripheral intravenous cannulation"[text word] OR "catheterization of vein and artery"[text word] OR "vascular cannulation"[text word] OR "cannulated vessels"[text word] OR "landmark techniques"[text word] OR cannula[text word] OR cannulae[text word] OR "nasal cannula"[text word] OR "Cannula"[Mesh] OR "Catheters"[Mesh] OR vein[text word] OR "Veins"[Mesh] OR insertion[text word] OR "vessel penetration"[text word] OR "skin penetration"[text word] OR "catheter redirection"[text word] OR "Vascular Access Devices"[Mesh] OR "Catheters"[Mesh] OR "Catheterization, Peripheral"[Mesh])))) AND ((("emergency department"[text word] OR "emergency services, hospital"[text word] OR "hospital emergency services"[text word] OR "services, hospital emergency"[text word] OR "Emergency Service, Hospital"[Mesh] OR "emergency units"[text word] OR "emergency medicine"[text word] OR "Medicine, Emergency"[text word] OR "Emergency Medicine"[Mesh])))) OR (((sonographers[text word] OR "emergency department sonographer"[text word] OR "emergency medicine nurse"[text word] OR "emergency department patient"[text word]))))) AND ((((("randomized controlled trial"[text word] OR "randomized controlled study"[text word] OR "Randomized Controlled Trial" [Publication Type] OR "Controlled Clinical Trial" [Publication Type])))) OR ((("prospective study"[text word] OR "studies, prospective"[text word] OR "study, prospective"[text word] OR "Prospective Studies"[Mesh] OR "prospective randomized study"[text word] OR "prospective observation study"[text word]))))	246
prospective study studies, prospective study, prospective	"Prospective Studies"[Mesh]	((("prospective study"[text word] OR "studies, prospective"[text word] OR "study, prospective"[text word] OR "Prospective Studies"[Mesh] OR "prospective randomized study"[text word] OR "prospective observation study"[text word]))) OR ((("randomized controlled trial"[text word] OR "randomized controlled study"[text word] OR "Randomized Controlled Trial" [Publication Type] OR "Controlled Clinical Trial" [Publication Type])))		
prospective randomized study	N/A			
prospective observation study	N/A			
randomized controlled trial	"Randomized Controlled Trial" [Publication Type] "Controlled Clinical Trial" [Publication Type]			
randomized controlled study				
			(((((((("long axis"[text word] OR "in-plane"[text word]) OR ("short axis"[text word] OR "out-of-plane"[text word]))))) AND (((ultrasound*[text word] OR ultrasonography[text word] OR "ultrasound imaging"[text word] OR "Imaging, Ultrasound"[text word] OR "Imagings, Ultrasound"[text word] OR "Ultrasound Imagings"[text word] OR "Sonography, Medical"[text word] OR "Medical Sonography"[text word] OR "Ultrasonography"[Mesh] OR "ultrasound-guided peripheral"[text word] OR "ultrasound-guided peripheral intravenous access"[text word] OR "ultrasound-guided vascular access"[text word] OR "ultrasound-guided venous access"[text word] OR USGVA OR "ultrasound-guided small vessel penetration"[text word] OR "ultrasound-guided small vessel penetration"[text word] OR "ultrasound guidance"[text word])))))) AND ((((("randomized controlled trial"[text word] OR "randomized controlled study"[text word] OR "Randomized Controlled Trial" [Publication Type] OR "Controlled Clinical Trial" [Publication Type])))) OR ((("prospective study"[text word] OR "studies, prospective"[text word] OR "study, prospective"[text word] OR "Prospective Studies"[Mesh] OR "prospective randomized study"[text word] OR "prospective observation study"[text word]))))	554
			Search (((((((("long axis"[text word] OR "in-plane"[text word]) OR ("short axis"[text word] OR "out-of-plane"[text word]))))) AND (((ultrasound*[text word] OR ultrasonography[text word] OR "ultrasound imaging"[text word] OR "Imaging, Ultrasound"[text word] OR "Imagings, Ultrasound"[text word] OR "Ultrasound Imagings"[text word] OR "Sonography, Medical"[text word] OR "Medical Sonography"[text word] OR "Ultrasonography"[Mesh] OR "ultrasound-guided peripheral"[text word] OR "ultrasound-guided peripheral intravenous access"[text word] OR "ultrasound-guided vascular access"[text word] OR "ultrasound-guided venous access"[text word] OR USGVA OR "ultrasound-guided small vessel penetration"[text word] OR "ultrasound-guided small vessel penetration"[text word] OR "ultrasound guidance"[text word])))))) AND ((((("randomized controlled trial"[text word] OR "randomized controlled study"[text word] OR "Randomized Controlled Trial" [Publication Type] OR "Controlled Clinical Trial" [Publication Type])))) OR ((("prospective study"[text word] OR "studies, prospective"[text word] OR "study, prospective"[text word] OR "Prospective Studies"[Mesh] OR "prospective randomized study"[text word] OR "prospective observation study"[text word]))))	1301
Scopus (All keywords searches)	Search Strategies	Final Search Strategies	Number of Search Results	
"in-plane"	TITLE-ABS-KEY(("long axis" OR "in-plane") OR ("short axis" OR "out-of-plane"))	( TITLE-ABS-KEY ( ( ( "long axis" OR "in-plane" ) OR ( "short axis" OR "out-of-plane" ) ) ) ) AND ( TITLE-ABS-KEY ( ( ultrasound* OR ultrasonography OR "ultrasound imaging*" OR "medical sonography" OR "ultrasound-guided peripheral" OR "ultrasound-guided peripheral intravenous access" OR "ultrasound-guided vascular access" OR "ultrasound-guided venous access" OR usgva ) ) )	2,499	
"out-of-plane"				
long axis				
short axis				
ultrasound*	TITLE-ABS-KEY(ultrasound OR ultrasonography OR "ultrasound imaging" OR "medical sonography" OR "ultrasound-guided peripheral" OR "ultrasound-guided peripheral intravenous access" OR "ultrasound-guided vascular access" OR "ultrasound-guided venous access" OR USGVA")			
ultrasonography ultrasound imaging Ultrasound Imagings Medical Sonography				
ultrasound-guided peripheral				
ultrasound-guided peripheral intravenous access				
ultrasound-guided vascular access				
ultrasound-guided venous access				
USGVA				
ultrasound-guided small vessel penetration				
ultrasound guidance				
peripheral IV	TITLE-ABS-KEY("peripheral IV" OR "peripheral intravenous cannulation" OR "catheterization of vein and artery" OR "vascular cannulation" OR "cannulated vessels" OR "landmark techniques" OR cannula OR cannulae OR "nasal cannula" OR vein OR insertion)	TITLE-ABS-KEY(("long axis" OR "in-plane") OR ("short axis" OR "out-of-plane")) AND TITLE-ABS-KEY(ultrasound* OR ultrasonography OR "ultrasound imaging*" OR "medical sonography" OR "ultrasound-guided peripheral"OR "ultrasound-guided peripheral intravenous access" OR "ultrasound-guided vascular access" OR "ultrasound-guided venous access" OR USGVA) AND TITLE-ABS-KEY("peripheral IV" OR "peripheral intravenous cannulation" OR "catheterization of vein and artery" OR "vascular cannulation" OR "cannulated vessels" OR "landmark techniques" OR cannula OR cannulae OR "nasal cannula" OR vein OR insertion)	281	
peripheral intravenous cannulation				
catheterization of vein and artery				
vascular cannulation				
cannulated vessels				
landmark techniques				
cannula cannulae nasal cannula				
vein				
insertion				
vessel penetration				
skin penetration				
catheter redirection				
emergency department hospital emergency services emergency units	TITLE-ABS-KEY("emergency department OR 'hospital emergency service*" OR "emergency unit*" OR "emergency medicine" OR sonographer* OR "emergency department sonographer" OR "emergency medicine nurse" OR "emergency department patient")	TITLE-ABS-KEY(("long axis" OR "in-plane") OR ("short axis" OR "out-of-plane")) AND TITLE-ABS-KEY(ultrasound* OR ultrasonography OR "ultrasound imaging*" OR "medical sonography" OR "ultrasound-guided peripheral"OR "ultrasound-guided peripheral intravenous access" OR "ultrasound-guided vascular access" OR "ultrasound-guided venous access" OR USGVA) AND TITLE-ABS-KEY("peripheral IV" OR "peripheral intravenous cannulation" OR "catheterization of vein and artery" OR "vascular cannulation" OR "cannulated vessels" OR "landmark techniques" OR cannula OR cannulae OR "nasal cannula" OR vein OR insertion) AND TITLE-ABS-KEY("emergency department OR 'hospital emergency service*" OR "emergency unit*" OR "emergency medicine" OR sonographer* OR "emergency department sonographer" OR "emergency medicine nurse" OR "emergency department patient")	17	
emergency medicine				
sonographers				
emergency department sonographer				
emergency medicine nurse				
emergency department patient				
				( TITLE-ABS-KEY ( ( ( "long axis" OR "in-plane" ) OR ( "short axis" OR "out-of-plane" ) ) ) ) AND ( TITLE-ABS-KEY ( ( ultrasound OR ultrasonography OR "ultrasound imaging" OR "medical sonography" OR "ultrasound-guided peripheral" OR "ultrasound-guided peripheral intravenous access" OR "ultrasound-guided vascular access" OR "ultrasound-guided venous access" OR usgva ) ) ) AND ( TITLE-ABS-KEY ( ( "emergency department OR 'hospital emergency service*" OR "emergency unit*" OR "emergency medicine" OR sonographer* OR "emergency department sonographer" OR "emergency medicine nurse" OR "emergency department patient" ) ) )	53	
"randomized controlled trial" "prospective stud*"	TITLE-ABS-KEY ( "randomized controlled trial" OR "prospective stud*" )					( TITLE-ABS-KEY ( ( ( "long axis" OR "in-plane" ) OR ( "short axis" OR "out-of-plane" ) ) ) ) AND ( TITLE-ABS-KEY ( ( ultrasound* OR ultrasonography OR "ultrasound imaging*" OR "medical sonography" OR "ultrasound-guided peripheral" OR "ultrasound-guided peripheral intravenous access" OR "ultrasound-guided vascular access" OR "ultrasound-guided venous access" OR usgva ) ) ) ) AND ( TITLE-ABS-KEY ( "randomized controlled trial" OR "prospective stud*" ) )	299	
